# Tumor-Immune Signatures of Treatment Resistance to Brentuximab Vedotin with Ipilimumab and/or Nivolumab in Hodgkin Lymphoma

**DOI:** 10.1158/2767-9764.CRC-24-0252

**Published:** 2024-07-15

**Authors:** Edgar Gonzalez-Kozlova, Hsin-Hui Huang, Opeyemi A. Jagede, Kevin Tuballes, Diane M. Del Valle, Geoffrey Kelly, Manishkumar Patel, Hui Xie, Jocelyn Harris, Kimberly Argueta, Kai Nie, Vanessa Barcessat, Radim Moravec, Jennifer Altreuter, Dzifa Y. Duose, Brad S. Kahl, Stephen M. Ansell, Joyce Yu, Ethan Cerami, James R. Lindsay, Ignacio I. Wistuba, Seunghee Kim-Schulze, Catherine S. Diefenbach, Sacha Gnjatic

**Affiliations:** 1 Department of Oncological Sciences, Icahn School of Medicine at Mount Sinai, New York, New York.; 2 Tisch Cancer Institute, Icahn School of Medicine at Mount Sinai, New York, New York.; 3 Precision Immunology Institute, Icahn School of Medicine at Mount Sinai, New York, New York.; 4 Department of Population Health Science and Policy, Icahn School of Medicine at Mount Sinai, New York, New York.; 5 Department of Data Science, Dana-Farber Cancer Institute, Boston, Massachusetts.; 6 Human Immune Monitoring Center, Icahn School of Medicine at Mount Sinai, New York, New York.; 7 Cancer Therapy Evaluation Program, Division of Cancer Treatment and Diagnosis, NCI, Bethesda, Maryland.; 8 CIMAC-CIDC Network, Pipeline Development and Portal Integration, Dana-Farber Cancer Institute, Boston, Massachusetts.; 9 Department of Translational Molecular Pathology, The University of Texas MD Anderson Cancer Center, Houston, Texas.; 10 Washington University School of Medicine, New York, New York.; 11 Mayo Clinic, New York, New York.; 12 Perlmutter Cancer Center, NYU Langone Health, New York, New York.

## Abstract

To investigate the cellular and molecular mechanisms associated with targeting CD30-expressing Hodgkin lymphoma (HL) and immune checkpoint modulation induced by combination therapies of CTLA4 and PD1, we leveraged Phase 1/2 multicenter open-label trial NCT01896999 that enrolled patients with refractory or relapsed HL (R/R HL). Using peripheral blood, we assessed soluble proteins, cell composition, T-cell clonality, and tumor antigen-specific antibodies in 54 patients enrolled in the phase 1 component of the trial. NCT01896999 reported high (>75%) overall objective response rates with brentuximab vedotin (BV) in combination with ipilimumab (I) and/or nivolumab (N) in patients with R/R HL. We observed a durable increase in soluble PD1 and plasmacytoid dendritic cells as well as decreases in plasma CCL17, ANGPT2, MMP12, IL13, and CXCL13 in N-containing regimens (BV + N and BV + I + N) compared with BV + I (*P* < 0.05). Nonresponders and patients with short progression-free survival showed elevated CXCL9, CXCL13, CD5, CCL17, adenosine–deaminase, and MUC16 at baseline or after one treatment cycle and a higher prevalence of NY-ESO-1-specific autoantibodies (*P* < 0.05). The results suggest a circulating tumor-immune-derived signature of BV ± I ± N treatment resistance that may be useful for patient stratification in combination checkpoint therapy.

**Significance:**

Identification of multi-omic immune markers from peripheral blood may help elucidate resistance mechanisms to checkpoint inhibitor and antibody–drug conjugate combinations with potential implications for treatment decisions in relapsed HL.

## Introduction

FDA-approved novel therapies have transformed the treatment options available for relapsed or refractory (R/R) Hodgkin lymphoma (HL). Brentuximab vedotin (BV), an anti-CD30 antibody–drug conjugate (ADC), was FDA approved in 2011 for patients with R/R HL who have undergone autologous stem cell transplant or multiple chemotherapy regimens, based on a complete response (CR) rate of 34% and an overall duration of response of 5.6 months (20.5 months in those with CR; ref. 1). Subsequently, in 2016, the PD1-targeting checkpoint inhibitors nivolumab and pembrolizumab were also approved for R/R HL. However, single-agent nivolumab has a CR rate of 14% to 16%, and a progression-free survival (PFS) of 15 months in patients with prior exposure to BV (2). In solid tumors, studies have shown that combining anti-CTLA4 treatment (ipilimumab) with PD1 blockade (nivolumab or pembrolizumab) can improve response rates in diverse types of tumors, at the cost of a higher rate of adverse events (AEs; refs. 3, 4).

The phase 1/2 study E4412 (NCT01896999) evaluated the safety and efficacy of single or dual checkpoint blockade with ipilimumab (I) and/or nivolumab (N) in combination with the antibody–drug conjugate BV in R/R HL patients after one or more lines of therapy, with adequate performance status and organ function (5). This combination was hypothesized to deplete CD30-expressing Hodgkin and Reed/Sternberg cells and to activate T effector cells to target Hodgkin and Reed/Sternberg cell killing and overcome therapeutic resistance. We reported a CR rate of 57% (95% CI, 34%–78%) for BV + I, 61% (36%–83%) for BV + N, and 73% (50%–89%) for BV + I + N arms (5). An increased number of grade 3 to 4 AEs was associated with treatment arms that included ipilimumab (43%–55%) when compared with the BV + N arms (21%). These promising results prompted an expansion to the planned phase 2 of this trial with a randomized comparison of BV + N *versus* BV + I + N, which recently completed adult enrollment. Molecular and cellular immune profiling of biomarkers that could explain differential response or survival to these ADC and CPI combinations has not been described to date.

To identify immune mechanisms associated with BV ± I ± N immunotherapy and biomarkers of resistance or AEs, which could guide future treatment decisions, we applied longitudinal immune monitoring and analysis of blood specimens collected during phase 1 throughout the course of treatment. Using cellular and molecular multi-omics, we examined peripheral markers for associations with clinical outcomes. We performed four different assays using peripheral blood plasma and mononuclear cells on specimens collected from 54 patients from the phase 1 component of this trial (19 in the BV + I group, 16 in the BV + N group, and 19 in the triplet group; ref. 5) including (i) Olink proximity extension assay to detect 92 soluble protein plasma analytes, (ii) ELISA Grand Serology to measure circulating plasma antibody titers against 20+ known tumor antigens, (iii) mass cytometry using time of flight (CyTOF) to assess peripheral blood cell composition, and cell surface activation/inhibitory marker expression, and (iv) Bulk Vβ TCR-seq to quantify T-cell immune repertoire diversity. Data from these assays was correlated to response rate (categorical, from imaging data, best achieved) and survival (for predictions at baseline only).

## Materials and Methods

### Clinical trial and biospecimens

**Figure 1:**
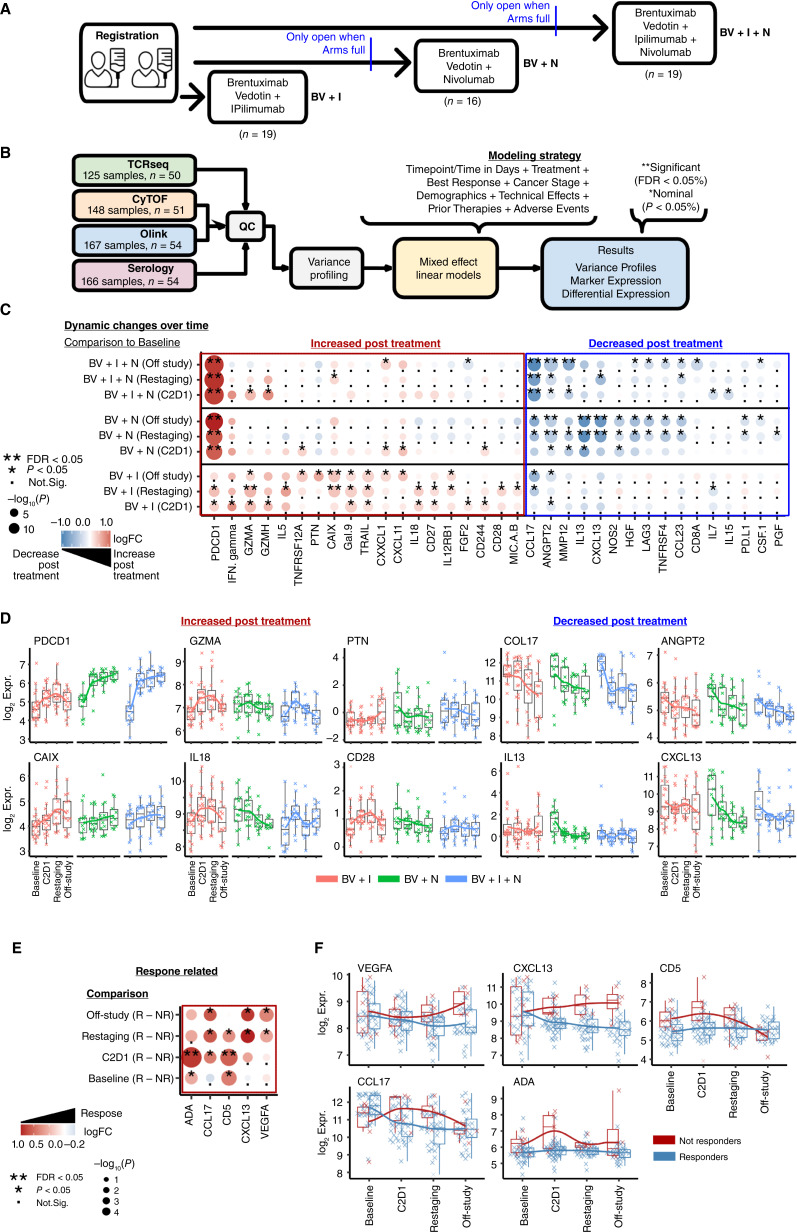
Protein dynamics in HL during checkpoint blockade treatment. **A,** Overview of the clinical trial E4412 experimental design. Three treatment arms included: (i) BV + ipilimumab (I), (ii) BV + nivolumab (N), and (iii) BV + I + N, with participant number (*n*) indicated. **B,** Regression modeling strategy using mixed effect models applied to analyze independently four different assay methodologies. Each assay was modeled considering relevant clinical variables and adjusted for multiple testing using FDR correction. **C,** Summary heatmap showing the log_2_-fold change (log_2_FC) between time points and treatments. The changes with a positive log_2_FC over time in color blue are associated with a decrease over time and red with an increase over time. The −log_10_ (*P* value) is represented by the size of the circles, indicating statistical significance as the circles increase. **D,** Line and boxplot figures showing the changes in expression for markers increased posttreatment such as PDCD1, GMZA, PTN, CAIX, IL18, CD28, and markers decreased posttreatment such as CCL17, ANGPT2, IL13, and CXCL13. **E,** Summary heatmap of differential expression associated with response. The changes with a positive log_2_FC in color blue are associated with a lower expression in nonresponders and red with higher expression in nonresponders. **F,** Line and boxplot examples of significant (*P* < 0.05 & FDR < 0.05) proteins associated with response (blue) or nonresponse (red) over time.

This clinical trial started with phase 1 and is currently completing phase 2 (ClinicalTrials.gov Identifier: NCT01896999). Patient characteristics, including demographics, previous lines of treatment, as well as safety and preliminary efficacy are described in ref. 5. During the dose escalation phase, three consecutive treatment groups were enrolled consisting of two arms receiving brentuximab vedotin 1.8 mg/kg q3w with ipilimumab at either 1 or 3 mg/kg q6w (BV + I, *n* = 6 for Arm A, *n* = 6 for Arm B); brentuximab vedotin at either 1.2 or 1.8 mg/kg q3w with nivolumab 3 mg/kg q3w (BV + N, *n* = 3 for Arm D, *n* = 6 for Arm E); and brentuximab vedotin at either 1.2 or 1.8 mg/kg q3w with nivolumab 3 mg/kg q3w and ipilimumab 1 mg/kg q12w (BV + I + N, *n* = 7 for Arm G, *n* = 5 for Arm H). Another seven patients per treatment group were subsequently enrolled into expansion arms (arms C, F, and I) at the highest respective doses to establish safety and preliminary efficacy (Fig. 1A). For this correlative study, Arms A-C (BV + I), D-F (BV + N), and G-I (BV + N) were respectively combined, as no significant clinical difference was observed related to dose escalation. Blood [cryopreserved after separation as plasma and peripheral blood mononuclear cells (PBMC)] was collected prior to the start of treatment (baseline), on day 1 of cycle 2 (prior to drug infusion; C2D1), at the time of first restaging PET/CT (±5 days) prior to cycle 4 (restaging) when clinical response was assessed, and after completion of therapy or off treatment (off-study). The best objective response rate, including CR and partial response, at each respective time point, was determined using the International Harmonization Project Group 2007 Revised Response Criteria according to Cheson and Deauville criteria as mandated by trial design (ref. 5; RRID:SCR_001905, RRID:SCR_015654, RRID:SCR_006442).

### Olink

Soluble protein analytes from peripheral blood plasma samples were quantified by Olink’s proximity extension immunoassay platform using the immuno-oncology panel. This multiplex immunoassay allows the simultaneous measurement of 92 proteins, including cytokines, chemokines, and immuno-oncology markers, across 96 plasma samples, including internal and external reference controls, and was performed following the manufacturer’s instructions (https://cimac-network.org). The Olink data were normalized into NPX values (Normalized Protein eXpression) on a log_2_ scale (https://www.olink.com/question/what-is-npx/; RRID:SCR_003899).

### Grand serology

ELISA was used to detect and quantify circulating IgG antibodies to known tumor antigens, as previously described (6). Briefly, plasma samples were analyzed by low-volume semi-automated ELISA for seroreactivity to a panel of recombinant protein antigens (NY-ESO-1, P53, SOX2, HORMAD1, ERG, DHFR, PRAME, WT1, MELAN-A, SURVIVIN, UBTD2, CT47, MAGE-A4, SSX4, CT10, SSX2, XAGE, GAGE7, and MAGE-A10). Low-volume 96-well plates were coated overnight at 4°C with 0.5–1 μg/mL antigen and blocked for 2 hours at room temperature with PBS containing 5% nonfat milk and 0.1% Tween 20. Plasma was titrated from 1/100 to 1/6,400 in 4-fold dilutions and added to blocked and washed 96-well plates. For assay validation and titer calculation, each plate contained positive and negative controls (pool of healthy donor sera). After overnight incubation, plates were extensively washed with PBS 0.2% Tween 20 and rinsed with PBS. Plasma antigen-specific IgG was detected after incubation with alkaline-phosphatase–conjugated goat anti-human IgG (SouthernBiotech 2040-4, diluted 1/4,500), revelation using AttoPhos substrate and buffer, and measurement using a fluorescence reader (BioTek Synergy). By linear regression, a reciprocal titer was calculated for each sample and for each antigen as the predicted or interpolated dilution value at which the titration curve meets a cutoff value (7). A positive significant result was defined as reciprocal titers >100 (RRID:SCR_019873).

### CyTOF

Mass CyTOF analyses were performed on PBMCs using a harmonized protocol as described previously (8). Briefly, 1–5 × 10^6^ thawed PBMCs were barcoded using palladium-based mass tags. Cells were then stained with a metal-conjugated antibody panel designed to characterize major immune subsets and surface activation markers, along with bead controls spiked in for data normalization. FCS files underwent bead-based normalization, followed by the exclusion of Ce140^+^ beads and bead-cell doublets, Gaussian ion cloud multiplet fusion events, and Rh103^+^ dead cells. Major immune cell subsets were identified using a hierarchical clustering approach (Astrolabe Diagnostics, Inc) and further confirmed using manual gating. The resulting tables contain cell number, cell frequency, and marker expression quantiles. Data were transferred to R for differential abundance and surface marker expression analysis using orloj, lme4, dream, and survival packages (RRID:SCR_021055, RRID:SCR_019916, RRID:SCR_019917, and RRID:SCR_021669). The panel of antibodies and reagents used for CyTOF are included in Supplementary Table S1.

### TCRseq

We used the immunoSEQ Kit from the Adaptive Biotechnologies Corporation that targets T-cell receptor beta chain (TCRβ) genes to enumerate rearranged TCRβ sequences in DNA isolated from PBMCs. The assay specifically targets the Complementarity Determining Region 3 (CDR3) of human TCRβ gene sequences, formed by rearrangement of the Variable (V), Diversity (D), and Joining (J) gene segments and including nontemplate Nucleotide (N) insertions and deletions at the gene segment junctions. Application of the immunoSEQ Kit was analytically validated and performed by the MD Anderson Cancer Center CIMAC. The minimum DNA input for the assay was 200 ng per sample. Specifically, the DNA was processed with immunoSEQ hsTCRB kit (cat # ISK10050) and Illumina MiSeq Reagent Kit v3 to generate libraries and sequenced by Illumina MiSeq Sequencing system (150 cycles). The resulting FASTQ files were processed with the Immunarch pipeline to obtain individual clonal quantifications. The resulting data was analyzed using R, lme4, dream, and survival packages (RRID:SCR_014709).

### Statistical analysis

#### Quality controls

The analysis for all datasets (Olink, Serology, CyTOF, and TCRseq) was performed in R software using a mixed linear model strategy to adjust for relevant clinical variables and demographics. The data distributions for markers and cell populations for all assays were investigated as part of routine quality control to identify biases and corrected as follows: (i) samples with more than 50% missing values in any analyte were excluded, (ii) Olink analytes that were less than the limit of detection in more than 50% of samples were excluded, and (iii) CyTOF cell populations unassigned by Astrolabe were ignored. QC analyses were used to identify biases such as low detection and poor-quality samples.

#### Variance analysis

Sample variance profiles were performed to assess the effect of covariates with assay data (Olink, Serology, CyTOF, and TCRseq) using the package variancePartition/Dream on R (9). Covariates with <5% effect on the model were excluded from modeling (RRID:SCR_001905, RRID:SCR_015654, and RRID:SCR_006442).

#### Survival and Cox proportional hazard models

Univariable and multivariable regression models were used to estimate the HRs and corresponding 95% confidence intervals for OS (overall survival) and PFS. Log-rank and Gehan–Breslow tests were used to assess the significance of the difference between endpoints for OS and PFS. The univariable models were used to determine which covariates should be kept in the multivariable models. Significance was defined as adjusted *P* values or FDR < 0.05.

#### Adjust *P* values for multiple comparisons

For multi-omic assays (Olink, Serology, and CyTOF), we applied moderate *t* test statistics. We adjusted *P* values using the Benjamini and Hochberg method (1995). This helps to control the FDR, the expected proportion of false discoveries among the rejected hypotheses. Nonetheless, throughout the manuscript, we show nominally significant results as *P* < 0.05 and adjusted *P* values represented as FDR < 0.05.

#### Differential expression

Differential protein expression analysis was performed in R using the packages dream and lme4 from bioconductor. The mixed effect models were built using the covariates shown in Fig. 1B. For Olink the independent variables were individual protein levels (NPX). For CyTOF, the independent variables were the surface markers with 95 quantile values. The results were visualized using pheatmap and ggplot2 packages.

#### Differential abundance

We used the limma-dream-lme4 pipeline (9) in R to assess differential abundance between populations while modeling the covariates previously indicated in Fig. 1B. This approach was used for CyTOF and TCRseq. The clonal expansion populations were defined by Immunarch (10). T-cell clones were classified into four groups: clones that had little evidence of expansion (unique/small clones/nonexpanded, 1e−5 < *x* ≤ 1e−4% of total clones), clones with some or medium expansion (1e−4 < *x* ≤ 0.001% of total clones), clones with large expansion (0.001 < *x* ≤ 0.01% of total clones), and hyperexpanded clones (0.01 < *x* ≤ 1% of total clones).

#### Correlation analyses

We used cor, corrplot, pvclust, and hmisc packages in R-Stats to perform Pearson (linear) and Spearman (nonlinear) correlations between analytes and endpoints.

#### Prediction of PFS using logistic regression

We used the package RMS and ROCR available on R to build classifiers of PFS status using the Olink analytes as predictors and clinical variables as covariates. The internal validation was done using cross-fold five validation.

### Data availability

All data is available upon request at the CIDC-CIMAC portal: https://cidc.nci.nih.gov/ upon request. All code used for analysis is available upon request at https://github.com/eegk. Data used to generate figures shown in this article are attached as Supplementary Table S2, including data for Olink, CyTOF populations, Serology, and TCR beta chain frequency.

## Results

Three consecutive treatment groups (BV + I, BV + N, or BV + I + N) were enrolled, representing 54 patients with available biospecimens evaluable for correlative markers (Fig. 1A). Blood samples were collected before treatment (baseline), during cycle 2 (C2D1), during patient re-evaluation (restaging), and after completion (off-study) to assess molecular and cellular baseline measurements and changes over time on all available PBMC and plasma samples (Supplementary Table S1). We performed assay-specific quality control and variance profiling followed by a linear mixed effect model to identify differential markers across time, treatments, and responses (Fig. 1B). This approach allows to minimize the effects of stage and tumor size (bulky disease; ref. 11). Statistical significance was defined as FDR adjusted or unadjusted *P* values.

### Dynamic changes in peripheral blood plasma soluble analytes associated with treatment benefit

Soluble protein analyte profiles were measured using a standardized panel of 92 inflammation and immuno-oncology-related proteins (Olink) in all 54 patients with available longitudinal plasma samples (Supplementary Table S1). First, we assessed significant changes from baseline related to treatment arms. Treatment with BV + I + N or BV + N led primarily to a durable increase in soluble PDCD1^∗^/PD1^∗^ levels, whereas BV + I induced increases from baseline for an array of T-cell effector and cytotoxicity-associated markers such as IFNγ, GZMA^∗^, GZMH, CD27, CD28, and IL12RB1 (^∗^FDR < 0.05 or *P* < 0.05; Fig. 1C and D; Supplementary Fig. S1). Treatments with BV + I also increased decoy and apoptotic markers CAIX^∗^, PTN, MICA/B, Gal9, and TRAIL (Fig. 1C; Supplementary Fig. S1), which was not observed after N-containing treatment. Conversely, levels of several circulating proteins associated with inflammation, including CCL17^∗^, ANGPT2^∗^, MMP12^∗^, IL13^∗^, CXCL13^∗^, and CCL23, were high at baseline and showed a decrease over time associated with N-containing treatments but less so with BV + I (Fig. 1C). In addition, T-cell survival- and exhaustion-related cytokines (LAG3, TNFRSF4/OX40, CD8A, IL7, IL15, and PDL1) were decreased after nivolumab use but not in BV + I (Fig. 1C). Overall, the largest change from baseline was observed for soluble PCDC1/PD1 levels in N-containing therapy groups, attributed in part to drug interaction in which nivolumab-bound PD1 may be stabilized in circulation (Fig. 1C and D). Still, even in the absence of nivolumab (BV + I group), soluble PD1 levels also increased from baseline to C2D1, suggesting immune activation.

Next, we asked whether soluble analytes differed per timepoint between responders and nonresponders, using the best overall response achieved. Out of 54 patients, 49 had evaluable clinical response data. Responders were defined as those experiencing CR or partial response (*n* = 43), whereas nonresponders had SD or PD (*n* = 6). Analysis of clinical outcomes associated with Olink data was assessed regardless of treatment group (BV + I, BV + N, BV + I + N) due to the low number of events per group. The levels of plasma CXCL13, CCL17, and VEGFA showed gradual decreases from baseline in responders, whereas they significantly increased in nonresponders over time (Fig. 1E and F). Additionally, responders had lower ADA (adenosine–deaminase) and CD5 levels at baseline and stayed low throughout treatment, whereas nonresponders had spikes in ADA and CD5 levels early on which normalized toward the end of the study (Fig. 1E and F).

**Figure 2:**
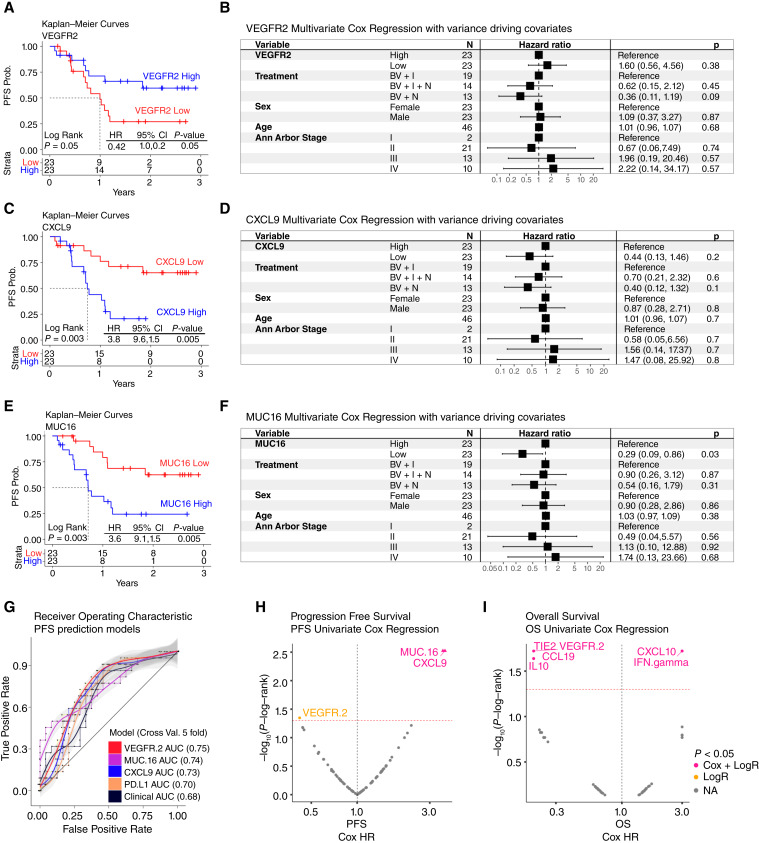
Association of plasma cytokines with clinical benefit. **A**, **C**, and **E,** PFS survival analysis shows the Kaplan–Meier curves for VEGFR2, CXCL9, and MUC16, respectively. Statistics using log-rank test and Cox proportional hazard models are shown. Higher than median VEGFR2 levels were associated with slower progression whereas higher than median levels of CXCL9 and MUC16 were associated with faster progression. **B**, **D**, and **F,** Forest plots for VEGFR2, CXCL9, and MUC16, respectively. Here, we show the multivariate statistics for each of these proteins including sex, age, treatment type, and cancer stage (Ann Arbor stage). These figures verify the directionality of the KM curves shown and show that these three proteins are independent of these clinically relevant covariates. **G,** Shows the receiver operating characteristic (ROC) curves for the prediction of PFS using VEGFR2, MUC16, CXCL9, PDL1, and clinical variables. Ordered from most relevant to least relevant model, reflected on the AUC values. **H,** Univariate PFS Cox modeling of Olink analytes. **I,** Univariate OS Cox modeling of Olink analytes. Both **I** and **H**, show on the *x*-axis the HR and the *y*-axis shows the −log_10_ (*P* values) based on the log-rank test.

To analyze the impact of soluble plasma analytes on clinical benefit, we performed univariate and multivariate Cox regression (adjusted for age, sex, tumor stage, and treatment group) and Kaplan–Meier analyses of PFS, using baseline Olink measurements. PFS benefit was associated individually with above median levels of VEGFR2 (Fig. 2A). Conversely, higher than median levels of CXCL9 and MUC16 were associated with worse PFS (Fig. 2C–E). Multivariate Cox regression confirmed MUC16 association with worse HRs, independently from age or sex or stage (Fig. 2F). However, CXCL9 and VEGFR2 only showed trends in multivariate Cox regression (Fig. 2B and D), potentially due to the effect of Ann Arbor Stage (Fig. 2B, D, and F).

To understand better the prognostic capabilities of these markers, we built a PFS classifier using clinical variables alone and combined with VEGFR2, MUC16, or CXCL9 (Fig. 2G). The results showed that indeed adding any of these three markers could predict progression better than clinical variables alone. Also, they showed that the area under the curve was the highest for VEGFR2 (0.75) followed by MUC16 (0.74), CXCL9 (0.73), PDL1 (0.70; selected as control), and clinical variables (0.68).

Next, we compared the log-rank test with Cox modeling in PFS (Fig. 2H), orthogonally verifying the results for VEGFR2, MUC16, and CXCL9. This approach was applied to overall survival revealing an association of IL10, CCL19, VEGFR2, and TIE2 with better OS outcomes and CXCL10 and IFN-gamma with worst OS outcomes (Fig. 2I). However, these findings were not reproduced in multivariate analysis (Supplementary Table S3).

In summary, we found potentially prognostic three markers associated with PFS outcomes, but we did not find a significant association with treatment, making it difficult to distinguish their predictive versus prognostic role.

### Dynamic changes in peripheral blood immune cell subsets associated with treatment benefit

**Figure 3:**
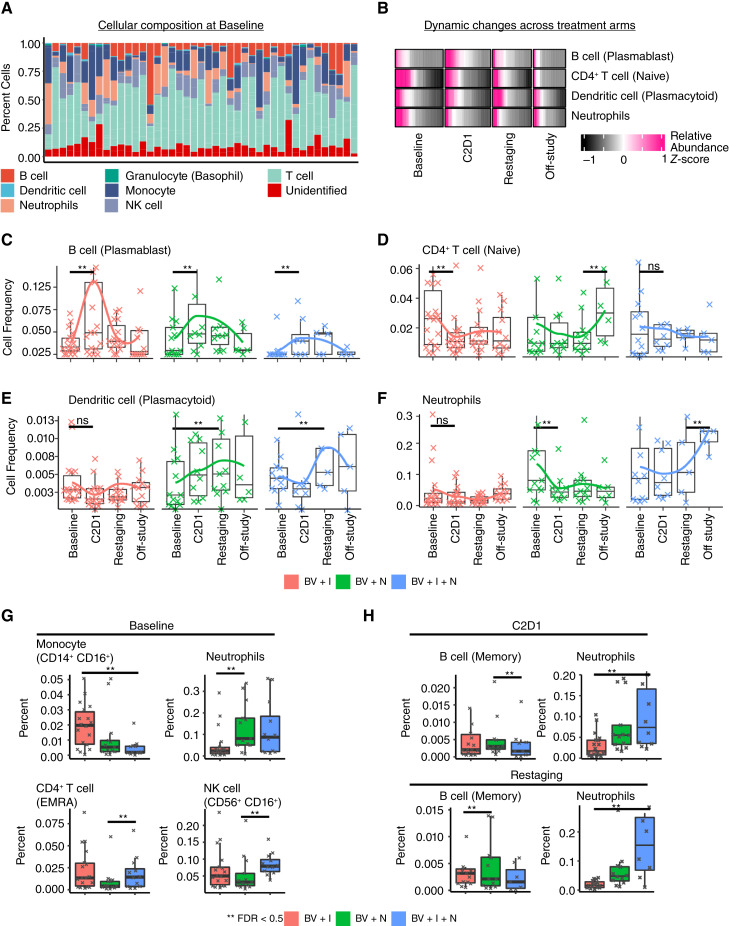
Cellular dynamics in HL during checkpoint blockade treatment. **A,** Cell type composition of major cell groups (myeloid and lymphoid) for all available samples at baseline. This bar plot shows the composition and variation of cellular components, including unassigned cells, across all patients. The cell types were identified using a semi-automated approach with the software Astrolabe. **B,** Heatmap map showing differentially abundant cell type changes over time. The rows are shown as relative abundance or scaled (*z*-score) allowing comparison across samples for each cell type simultaneously. **C–F,** Boxplots and line plots colored based on treatment showing the dynamics for the differentially abundant cell types. **G,** Boxplots showing the differentially abundant cells between treatments at baseline. **H,** Comparison of cell abundances for different treatments after the start of treatment.

PMBC-derived subpopulations were quantified using CyTOF from 51 patients with available cryopreserved biospecimens (Supplementary Table S1). Differences in 30 immune cell subsets and eight compartments (including a category for unidentified cells) were quantified simultaneously by semi-automated analysis using the Astrolabe and R platforms. The predominant immune compartments in blood were T cells, followed by neutrophils, monocytes, and B Cells (Fig. 3A). Treatments with nivolumab led to an increase in plasmacytoid dendritic cells (pDC) in the bloodstream (FDR < 0.05; Fig. 3B–E). Plasmablast B cells also showed a transient increase from baseline across all three therapy groups, with the most significant increase observed in BV + I after the initial treatment cycle (Fig. 3B and C). Additionally, neutrophils and naïve CD4^+^ T cells showed significant treatment-dependent but divergent changes from baseline, occurring in BV + N *versus* other combinations (Fig. 3D–F). Specifically, naïve CD4^+^ T cells decreased after one cycle of BV + I but increased at the end of BV + N, whereas neutrophils decreased after one cycle of BV + N but increased at the end of BV + I + N (Fig. 3D–F). When comparing treatment groups per timepoint, differences in cellular abundance were found at baseline in CD14^+^ CD16^+^ monocytes, neutrophils, CD4^+^ T_EMRA_ cells, and CD56^+^ CD16^+^ NK cells (Fig. 3G), pointing to potential imbalances prior to treatment in these nonrandomized patients. Posttreatment memory B cells were significantly more abundant in BV + N *versus* others, whereas neutrophils were more frequent in BV + I + N *versus* others (Fig. 3H). Overall, nivolumab-containing regimens seemed to significantly raise levels of antigen-presenting cells (pDCs, Fig. 3E–H), whereas the triplet combination resulted in higher inflammatory cell subsets (neutrophils, Fig. 3F–H).

### Differential expressions of peripheral immune cell surface markers associated with treatment benefit

PBMC subsets were also evaluated by CyTOF for inducible surface markers and changes in their expression. Durable decrease in PD1 expression in various T-cell subsets (CD8^+^ & CD4^+^) was seen after nivolumab treatments compared with BV + I (Supplementary Fig. S2A and S2B), attributed to known masking of epitope accessibility after nivolumab administration, which prevents PD1 detection during the assay, rather than to a biological observation. Reduced expression of other cell surface markers was associated with nivolumab treatments relative to ipilimumab, including HLA-DR (on memory B cells, CD4^+^ CD8^+^ T cells), CD45RA (on memory B cells), CD39 (on memory B cells, CD27^−^ B cells), CD8 (on CD56^+^ CD16^+^ NK cells), CD57 (on CD56^+^ CD16^−^ NK cells, type 2 CD1c^+^ dendritic cells), and CD95 (on CD14^−^ CD16^+^ monocytes; Supplementary Fig. S2A and S2B).

When analyzing surface expression changes by response to treatment, CD56 and CD45 levels on NKT cells were found lower in nonresponders at baseline (Supplementary Fig. S2C and S2D). Similarly, CD57 expression on CD8^+^ T_EMRA_ started lower and increased in nonresponders over time (Supplementary Fig. S2C and S2D). Interestingly, pharmacodynamic changes posttreatment in CXCR3 expression on pDC showed higher expression in responders compared with nonresponders (Supplementary Fig. S2C and S2D). In summary, CXCR3 could be useful as an activation marker on pDC in responders, whereas T_EMRA_ expression of CD95 and CD57 was associated with resistance.

### Circulating antibodies to tumor-associated antigens associated with treatment benefit

**Figure 4:**
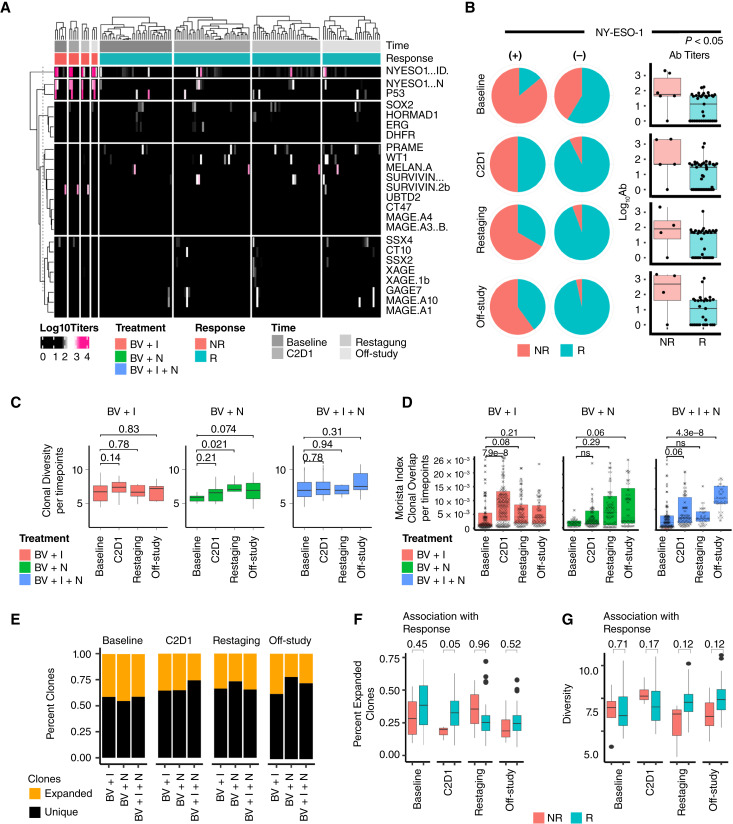
Cancer antigen detection and T-cell clonal dynamics associated with treatment and response. **A,** Heatmap showing cancer antigen detection by ELISA for all samples. The color represents the log_10_ scale antibody titers. Positive detection is considered above levels of 2 (pink), whereas negative detection is represented by the color black. The top rows of the figure show the treatment group, best overall response, and time. The figure is separated into nonresponders (left) and responders (right). Each column box is hierarchically clustered for simplicity. **B,** Pie charts showing the association of NY-ESO-1 presence (left column) and nondetection (middle column). Boxplots (right column) show all titer levels for nonresponders and responders. **C,** Boxplots showing the standardized absolute clonal diversity index calculated using Immunarch R package. The clonal diversity changes are shown over four time points (Baseline, C2D1, Restaging, and off-study) per treatment arm. The *P* value from the Wilcoxon rank test is shown on top of the boxes. The *y*-axis is identical for all three treatment arms. There was no statistical difference between the treatments due to the observed large variances (patient heterogeneity). **D,** Boxplots showing an increase in clonal overlap over time for all three treatments. *P* values were estimated using the Wilcoxon rank test. **E,** Clonal expansion was stratified into unique and expanded clones (black and orange, respectively). The stacked barplot shows the average percent of each clonal expansion class over time and per treatment arm. There were no statistical differences identified between treatment arms. **F,** Boxplots showing the percent abundance of only expanded clones shown for responders and nonresponders over time. There were no significant differences between these two groups except for C2D1 using the Wilcoxon rank test. **G,** Clonal diversity boxplots comparing responders and nonresponders. Overall, responders had a higher diversity, yet it did not reach statistical significance.

Autoantibody (AuAb) profiling of common tumor-associated antigens was performed in longitudinal plasma samples from all 54 patients using ELISA Grand Serology for IgG titers against a series of 19 full-length recombinant proteins (Supplementary Table S1). At multiple time points in each therapy group, tumor-associated antibodies were detected in responders and nonresponders (Fig. 4A). NY-ESO-1 AuAbs were detected in more than 40% of patients who were nonresponders, from baseline and at all four time points (Fig. 4B). In comparison, although prevalent at baseline, NY-ESO-1 AuAbs were absent in more than 90% of responder patients after treatment initiation. Antibody titers for NY-ESO-1 were more often not detected (negative) and had lower average titers in responders than nonresponders at all time points (Fig. 4B).

### T-cell clonal expansion association with treatment benefit

T-cell clones derived from PBMCs TCR Vβ were classified into two groups: clones that had little evidence of expansion (unique/small clones/nonexpanded, ≤1e−4% of total clones) and clones with evidence of expansion (>1e−4%). We used two standardized metrics (clonal expansion and diversity) for investigating the association of clonality with treatment or response. There were no significant differences in clonal diversity over time except an increase in BV + N at restaging compared with baseline (*P* < 0.05; Fig. 4C). However, clonal overlap increased during treatment regardless of treatment type (*P* < 0.05; Fig. 4D). There was no significant difference between clonal expansion between BV + I + N, BV + N, and BV + I (Fig. 4E). When looking at clinical benefit, the percentage of expanded clones was higher in responders versus nonresponders at C2D1 (*P* < 0.05) but not at other time points (Fig. 4F). Finally, clonal diversity was increased in responders compared with nonresponders at restaging and off-study, which approached but did not reach statistical significance (*P* > 0.05; Fig. 4G). Overall, evidence of clonal expansion and clonal overlap following treatment was found, with marginal contribution to clinical benefit.

### Biomarkers association with AEs

Although variations in the number of AEs were reported among BV + I + N, BV + N, and BV + I treatments, our analysis did not reveal a significant association between AEs and biomarkers in any of the assays. We meticulously excluded unlikely related and unrelated AEs, focusing on grades 3 to 5 and dose-limiting toxicities with the greatest clinical impact. The complexity of associating AEs with biomarkers stems from intricate interactions involving genetic predispositions, environmental influences, and individual response variations.

## Discussion

In this study, we examined peripheral molecular and cellular markers for their ability to distinguish differential treatment response and PFS in patients with R/R HL treated with a combination of single or dual checkpoint inhibitor with ADC (Supplementary Fig. S3). Our findings contribute to the understanding of the immune landscape of HL using rigorously validated and harmonized multi-omics technological platforms for immune monitoring of novel therapies. The overarching goal of these research efforts was to identify potential immune signatures for risk stratification and therapeutic decision-making for patients with HL treated with immunotherapy (12). Soluble plasma or serum proteins have previously been reported as capable of distinguishing HL from healthy patients through immune response-related markers such as PDL1, CCL17, CCL3, IL13, MMP12, TNFRS4, and LAG3 (13). We found proteins that increased in plasma posttreatment, particularly enriched in cytotoxicity-related markers (IFNγ, GZMA/H, and CD244) following treatment with I-containing arms, whereas decreases in stromal-derived factors, such as CCL17, ANGPT2, IL13, and CXCL13 were observed in N-containing arms. Interestingly, higher levels of CCL17, as well as ADA, CXCL13, CD5, and VEGFA, were associated with a lack of treatment response, regardless of treatment type. Some of these proteins have been previously associated with adverse HL outcomes: CCL17 from tumors, also known as TARC (14, 15); CXCL13 in PD1+ T cells (16); and VEGF in tumors (17, 18). Furthermore, elevated levels of these proteins have been linked to HL compared with healthy controls (19). Despite constitutive expression of PDL1 in HL and reports of serum PDL1 as a potential predictor of response (11, 20, 21), we did not observe soluble PDL1 as a clinically relevant marker in plasma. Interestingly, the strongest markers of progression were ADA and CD5, which were transiently elevated early posttreatment (C2D1) in nonresponders. Although these markers had not been previously shown as prognostic in HL, there is literature showing ADA and its ligand CD26 as higher in ALK-positive NHL and HL (22) as well as being associated with poor outcomes in other tumor types (23).

Importantly, despite high CR rates, many patients recur, and therefore, PFS may be a better prognostic marker of durable benefit. We identified elevated plasma CXCL9 and MUC16 at baseline and reduced VEGFR2 as associated with worse PFS. MUC16, also known as CA125, has been extensively described as a marker associated with progression in solid tumors (24), but it is underexplored in HL. The amount of soluble VEGFR2 may contribute to how much ligand is available for tumor growth and vascularization. It is also not clear why CXCL9 levels, which increase with immunotherapy, had a negative impact on PFS, but it could reflect patients with higher prior lines of treatment, because CXCL9 levels are affected by prior immunotherapies, or represent higher baseline inflammation, which has been described to be a poor predictor (25). Lymphoma cytogenetic features, including tumor mutation burden, could also affect the analytes measured in blood, but unfortunately, data from tumor tissue was not available for our analysis at the current time. Overall, our study validates previous studies and suggests novel soluble proteins associated with treatment resistance.

Although the potential role of CD4^+^ T cells as inflammatory/immune regulators in HL has previously been associated with response (26, 27), we found no changes in effector or regulatory CD4^+^ T cells except CD4^+^ T_EMRA_ and NK cells differentially prevalent at baseline across treatment groups. Additionally, increased B memory cells and neutrophils were associated with treatment (highest in BV + I + N), both of which have been associated with refractory disease (27). Interestingly, pDCs were generally highly increased with all treatments, and CXCR3 induction on pDC was associated with a favorable response to treatment. Although pDCs are generally rare, they are usually reliably identified due to their distinct lineage markers. Because CXCR3-ligands CXCL9 and CXCL10 were detected in the circulation of patients with poor survival, we speculate that they may reduce CXCR3 pDC from circulation due to homing to tissues. In contrast, CXCR3 pDC in blood would be expected to be more prevalent with low CXCL9/10. The data may indicate that pDCs have a pathogenic role in HL, as has been previously observed with increased circulating pDCs with favorable response to treatment of HL (28).

Patients with resistance to treatment also had increased levels of surface markers CD56 on NKT, CD57, and CD95 on CD8^+^ T_EMRA_, which may indicate improper differentiation of effectors. CD57 has been associated with terminal differentiation and senescence of NK cells, and our data suggests the expansion of this phenotype over time in nonresponders. Although provocative, these observations require prospective validation (29–32). T-cell clonal expansion is widely reported as a prognostic signature of response in patients with HL (33, 34), specifically when associated with the expansion of CD4^+^ T cells or gamma delta T cells (35). Patients with clonally expanded T cells at baseline confirmed some of these observations, and we also observed trends of increase in clonal diversity over time, although it did not reach statistical significance potentially due to patient heterogeneity.

Our study also investigated the impact of tumor-specific autoantibody (AuAb) profiles on drug mechanisms and outcomes. NY-ESO-1, MAGEA4, PRAME, and SSX2 are potential cancer–testis antigens that have been associated with HL in various studies and tested in clinical trials (36). Evidence in solid tumors suggests that patients with NY-ESO-1 preexisting immunity fare better than NY-ESO-1 seronegative patients after checkpoint blockade (37). Here, we observed the opposite, with nonresponders enriched in NY-ESO-1 Ab at baseline (Fig. 4A), although a small fraction (<15%) of responders also showed the presence of NY-ESO-1 AuAb (Fig. 4B). Like other cancer–testis antigens, NY-ESO-1 expression in cancer is induced by DNA hypomethylation and histone acetylation (38). Antibodies could therefore be a surrogate for more aggressive tumors, which we could not confirm due to the absence of tissues to correlate antigen presence. Although past studies have failed to link clinical benefit to the expression of these cancer–testis antigens in HL (39), more recent attempts at harnessing T-cell response via adoptive transfer have demonstrated safety and preliminary efficacy of targeting cancer–testis antigens (40). Therefore, considering the role of endogenous immunity using cancer-related plasma circulating AuAb could be useful and would be warranted in future studies.

Important limitations of this study include the absence of available tumor tissues to investigate the source or impact of peripheral markers on the tumor microenvironment. In addition, it is important to note that patients were not randomly assigned to treatment groups and that attrition of available samples occurred with time. Nevertheless, the statistical modeling strategy used allows minimization of these biases by incorporating fixed and random effects. Additionally, the large imbalance in responders *versus* nonresponders precluded treatment-specific analyses of clinical benefit, which were only evaluated for the entire cohort. Finally, we could not properly quantify neutrophil counts, known to be prognostic in HL, because cellular assays were conducted with PBMCs, though qualitative differences could still be assessed in neutrophils surviving density gradient purification.

In summary, we found that elevated circulating plasma proteins CXCL13, ADA, CXCL9, MUC16, and CCL17 as well as NY-ESO-1 autoantibodies were associated with poor outcomes to treatment with BV combined with I, N, or both. Together, it is possible that elevated baseline levels of plasma CXCL9, the presence of tumor-related NY-ESO-1 autoantibodies, and reduced plasma VEGFR2 highlight heavily pre-treated tumors that may exhibit primary resistance to treatment despite the presumed presence of immune infiltration and recognition. In addition, markers increasing from baseline in patients progressing through treatment include CXCL13, CCL17, and reduced clonal T-cell diversity, likely reflecting increasing tumor burden and activation of a Tfh axis previously associated with poor prognosis of lymphocyte-rich HL (16). Reduced cytotoxicity-related markers on NKT and T_EMRA_ were also seen at the start of treatment in patients with poor outcomes, whereas increases in circulating CXCR3 pDCs were associated with favorable response, as also observed independently (28). These results suggest drug-related mechanistic effects on immune cell activity that could contribute to treatment sensitivity or response versus resistance, and potentially impact treatment decision-making. If validated these findings may also suggest novel therapeutic strategies. The phase 2 component of this clinical trial (NCT01896999) has concluded enrollment, and we will prospectively validate the immune markers identified in this study. If validated, these may be important tools for a personalized approach to immunotherapy in HL.

## Supplementary Material

Figure S1 Supplemental Figure 1. A. Volcano plots showing differentially expressed proteins with P<0.05 between treatments.

Figure S2 Supplemental Figure 2. Cellular marker dynamics in Hodgkin lymphoma during checkpoint blockade treatment. A. Heatmap showing markers and cell types identified through CyTOF (cytometry using time of flight) that significantly change over time. The color indicates the standardized Log2FC in protein expression (Z-Score), and the size of the circles indicate the percent of cells expressing the marker. A dendrogram of the markers sorted hierarchically is shown on the right side. B. Line plots highlight the temporal changes for each of the markers shown in A. The color separates the values per treatment. C. Heatmap showing markers and cell types significantly associated with R (Responder) and NR (Not responder). D. Regression lines for examples of markers and cell types shown in C, separating response (R) and non-response (NR).

Figure S3 Supplemental Figure 3. Graphical abstract. Top boxes show a simplified trial design including 3 treatments composed by the combination of BV with I, N and I+N. We investigated the differences between responders and non-responders using standardized assays through the CIMAC’s network (ELISA, Olink, CYTOF and TCRseq). Bottom boxes summarize the main findings associated with specific treatments and treatment associated response or resistance.

Supplementary Table 1 CyTOF antibody panel details.

Supplementary Table 2 Experimental data userd in the article including Olink, CyTOF populational frequencies, Serology and TCRseq beta chain.

Supplementary Table 3 OS Multivariate analysis showing that markers significant during univariate analysis are not significant during multivariate analysis.
