# Supplementary figures and images for: Tumor-Immune Signatures of Treatment Resistance to Brentuximab Vedotin with Ipilimumab and/or Nivolumab in Hodgkin Lymphoma
Source: Cancer Res Commun. 2024 Jul 15;4(7):1726–37. doi: 10.1158/2767-9764.CRC-24-0252 (PMC11247952; doi:10.1158/2767-9764.CRC-24-0252)

# Supplemental Figure 1

A

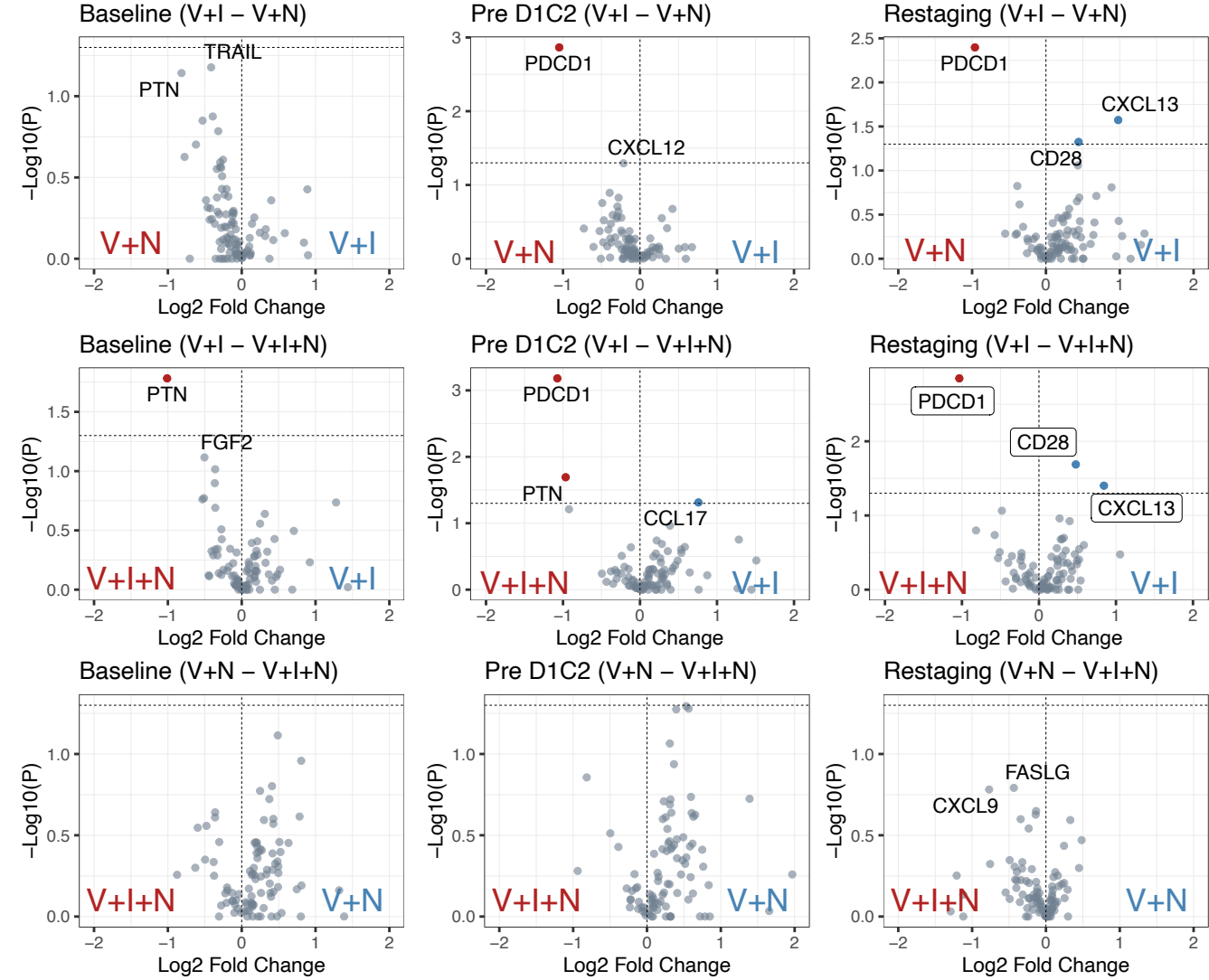

Supplement: Figure S1 — Supplemental Figure 1. A. Volcano plots showing differentially expressed proteins with P<0.05 between treatments. [file crc-24-0252_figure_s1_supps1.pdf]

# Supplemental Figure 2

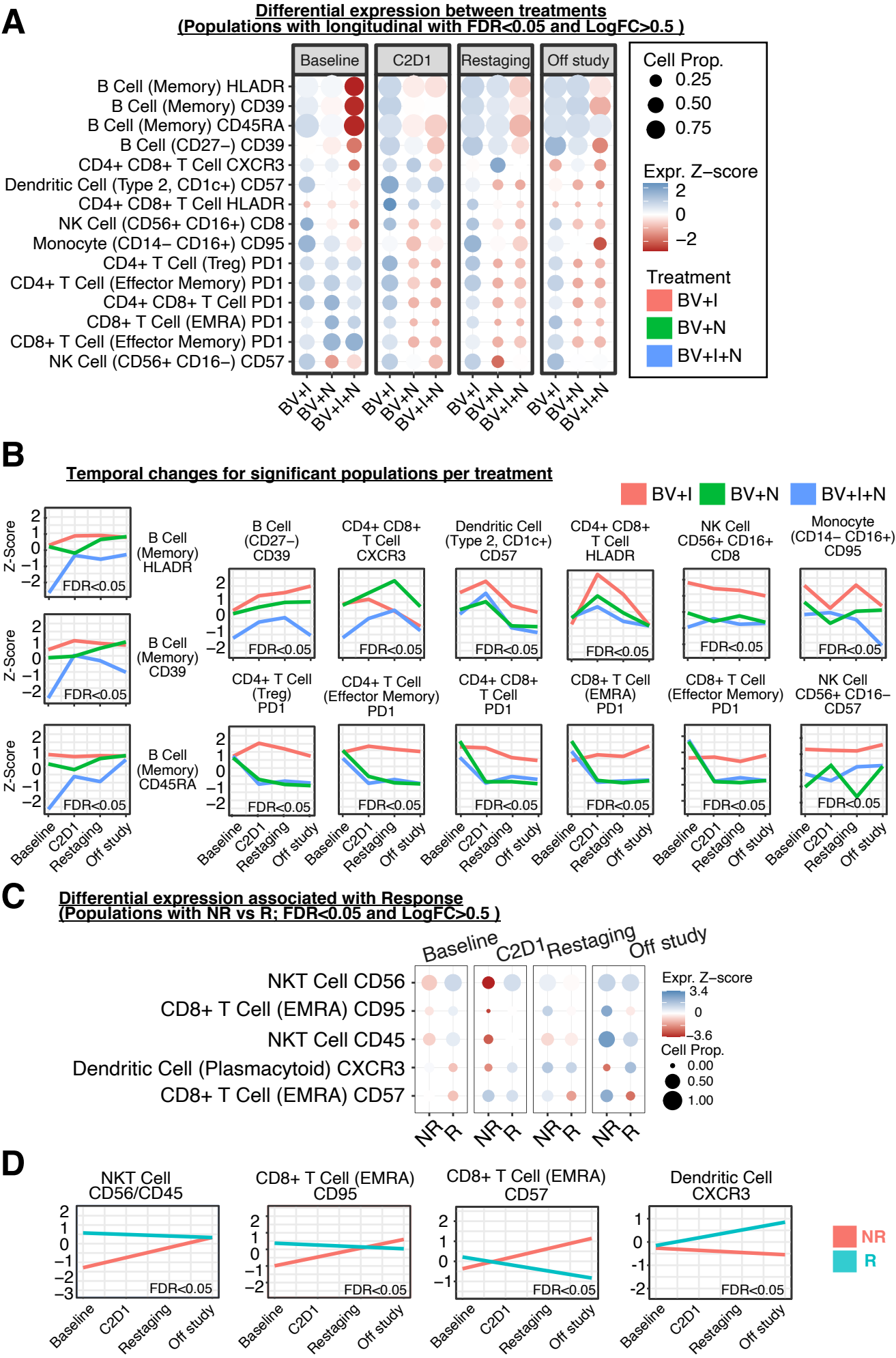

Supplement: Figure S2 — Supplemental Figure 2. Cellular marker dynamics in Hodgkin lymphoma during checkpoint blockade treatment. A. Heatmap showing markers and cell types identified through CyTOF (cytometry using time of flight) that significantly change over time. The color indicates the standardized Log2FC in protein expression (Z-Score), and the size of the circles indicate the percent of cells expressing the marker. A dendrogram of the markers sorted hierarchically is shown on the right side. B. Line plots highlight the temporal changes for each of the markers shown in A. The color separates the values per treatment. C. Heatmap showing markers and cell types significantly associated with R (Responder) and NR (Not responder). D. Regression lines for examples of markers and cell types shown in C, separating response (R) and non-response (NR). [file crc-24-0252_figure_s2_supps2.pdf]

# Supplemental Figure 3

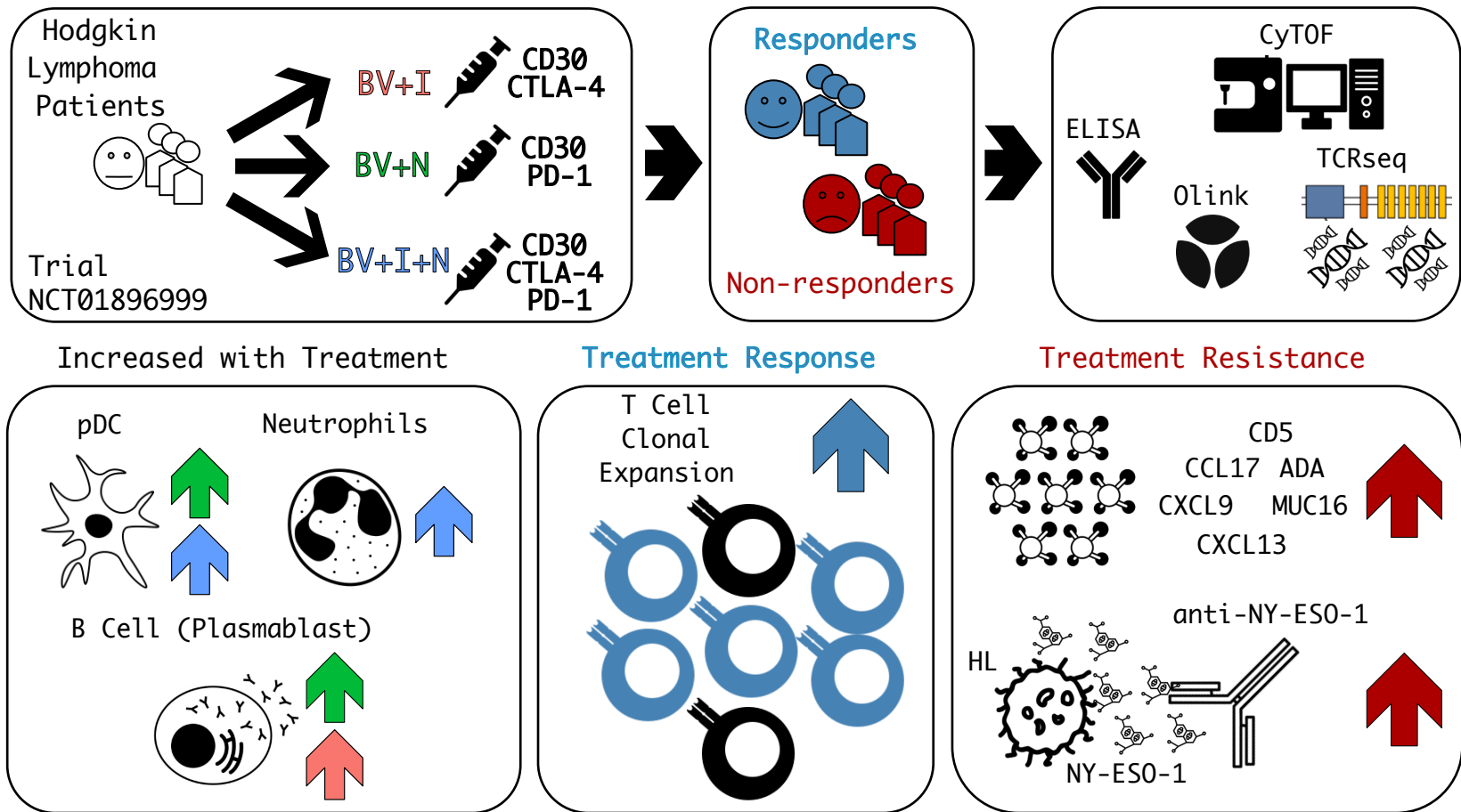

Supplement: Figure S3 — Supplemental Figure 3. Graphical abstract. Top boxes show a simplified trial design including 3 treatments composed by the combination of BV with I, N and I+N. We investigated the differences between responders and non-responders using standardized assays through the CIMAC’s network (ELISA, Olink, CYTOF and TCRseq). Bottom boxes summarize the main findings associated with specific treatments and treatment associated response or resistance. [file crc-24-0252_figure_s3_supps3.pdf]
